# Supplementary material for: A hollow TFG condensate spatially compartmentalizes the early secretory pathway
Source: Nat Commun. 2025 Apr 19;16:3715. doi: 10.1038/s41467-025-59118-1 (PMC12009431; doi:10.1038/s41467-025-59118-1)
Supplement: Supplementary file 1 — Supplementary Information [file 41467_2025_59118_MOESM1_ESM.pdf]

Supplementary Figures

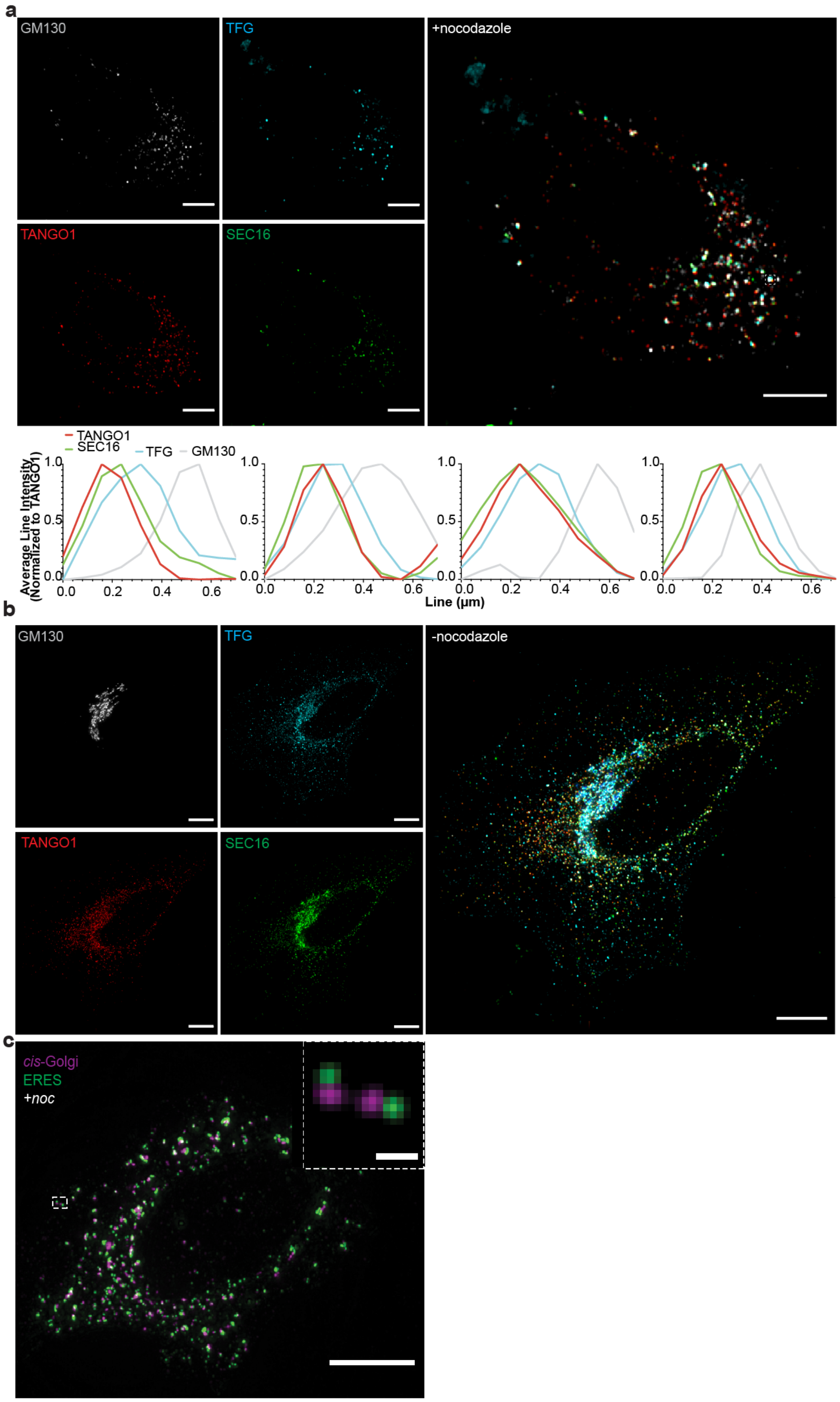

Supplementary Fig. 1 Localization of Sec16 and TFG at the ER-Golgi interface (cell overview for magnification provided in Fig. 1 a Micrographs of HeLa cells treated with nocodazole **a**

(+noc) or **b** (-noc), transfected with mGFP-Sec16L (green), FLAG-TFG-SNAP (cyan) and immunolabeled for endogenous GM130 (gray) and TANGO1 (red), Scale bar 10 µm. Individual line scans of ER-Golgi units used for averaged line scan (Fig. 1b) are given. **C** Micrograph of HeLa cells immunolabeled with ERES (green) and cis-Golgi (magenta) markers (max. intensity Z-projections). The Golgi ribbon was unlinked using nocodazole to facilitate visualization of individual ER-Golgi interfaces (+noc). Scale bar 10 µm, inset 500 nm. Source data are provided as a Source Data file.

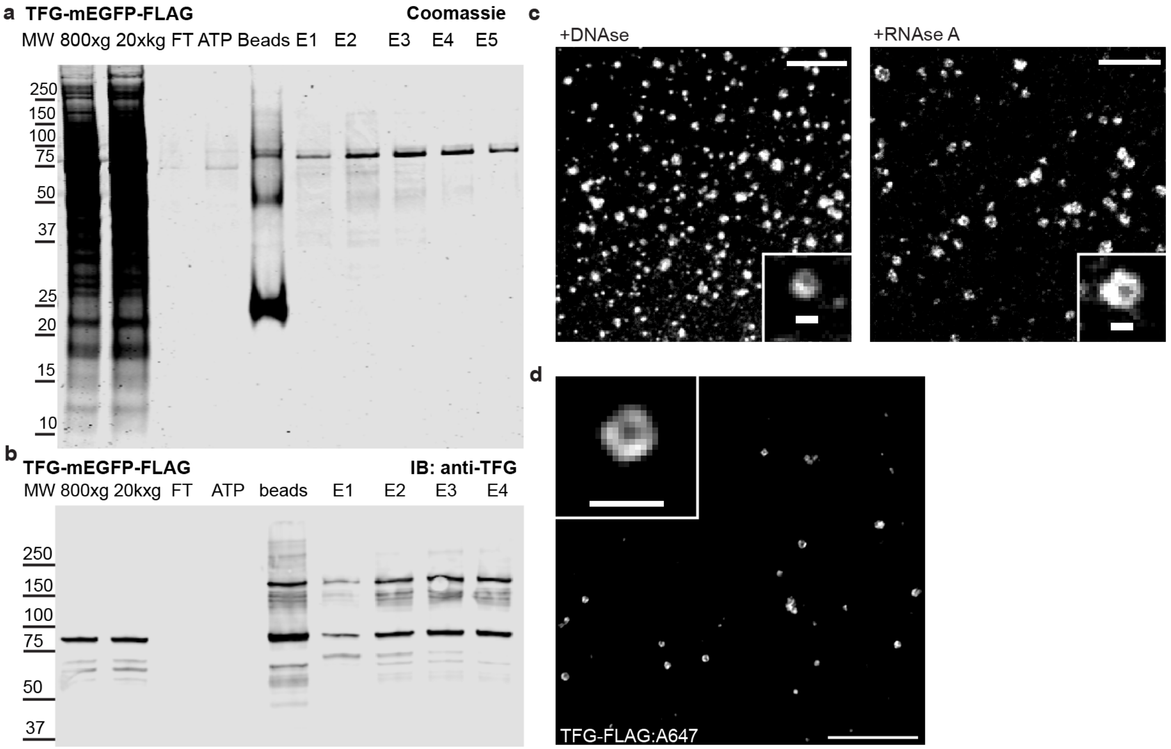

Supplementary Fig. 2 TFG purifies without significant contaminants from endogenous TFG, DNA, or RNA a Representative purification overview of TFG-mEGFP-FLAG purification from Expi293F suspension cells (Coomassie staining). Peak elution purity > 95%. **B** Anti-TFG Western blot of TFG-mEGFP-FLAG purification from Expi293F suspension cells. **C** TFG-162-240-mEGFP-FLAG lumen-containing condensates were treated with 1 U DNAse and imaged after a 3 minute incubation period. TFG-162-240-mEGFP-FLAG was purified in the presence of 10 U RNAse A (added during a wash step). (HEPES/KOH pH 7.3; 150 mM KCl; 20% (v/v) PEG 8 kDa). Scale bars 5 µm, 500 nm insets. **D** Confocal micrographs of condensates of recombinant TFG-FLAG fluorescently labeled with Alexa 647. Scale bar 5 µm, inset 500 nm. Source data are provided as a Source Data file.

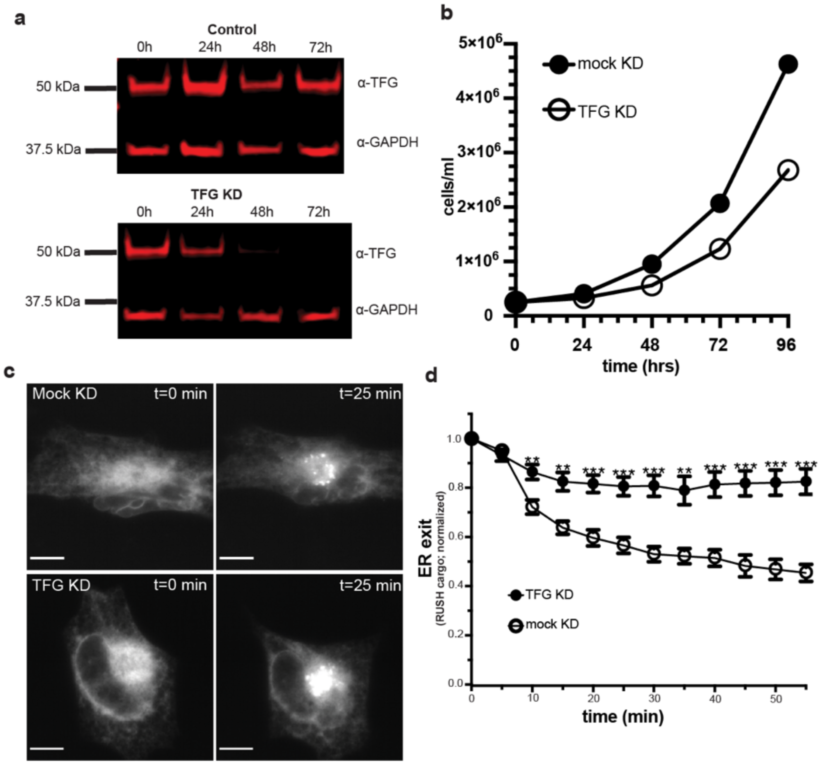

Supplementary Fig. 3. TFG impacts cargo export and cell growth. **A** Western blot of TFG siRNA knockdown (KD) and mock knockdown at 24, 48, and 72 hours. **B** Growth curve of TFG knockdown and mock knockdown Expi293F cells. **C** Live-cell visual correlates for RUSH assay. Cells were either subjected to mock or TFG siRNA treatment for 72 h and transfected with Str-KDEL_SBP-EGFP-Ecadherin. Cargo waves triggered upon incubation with biotin; scale bar 10 µm. **d** Quantification of ER exit of RUSH cargo (Ecadherin) in live cells for TFG knockdown (72 h; n = 9) and mock knockdown (72 h; n = 8) cells. Error bars represent standard deviation. Two-tailed unpaired t-tests (t = 5: p = 0.5532, t = 10: p = 0.0045, t = 15: p = 0.0013, t = 20: p = 0.0004, t = 25: p = 0.0003, t = 30: p = 0.0001, t = 35: p = 0.0014, t = 40: p = 0.0003, t = 45: p = 0.0002 , t = 50: p = 0.0001, t = 55: p < 0.0001). Source data are provided as a Source Data file.

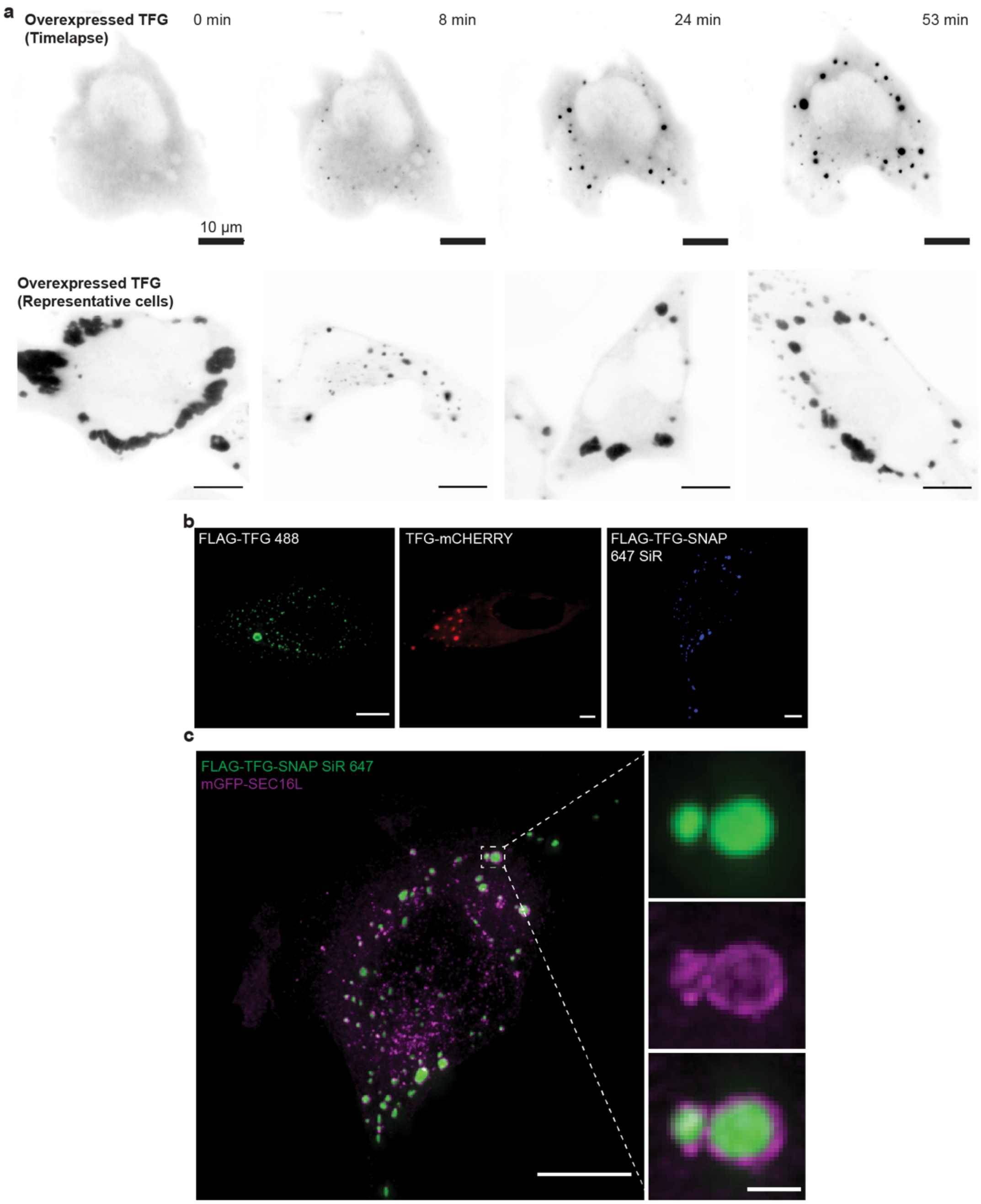

Supplementary Fig. 4. TFG condensates formed from overexpression are observed with a variety of tags and recruit Sec16 to their surface. A Top panel: Live-cell imaging of Hela cells transfected with TFG-mEGFP-FLAG 24 h post-transfection (Z-slice, intervals chosen to show nucleation and growth). Bottom panel: Representative images of TFG-mEGFP-FLAG overexpressed in HeLa cells exhibiting various condensate morphologies. **B** Left panel: cells transfected with FLAG-TFG immunostained with anti-TFG, Alexa Fluor 488 and imaged with widefield deconvolution microscopy. Middle panel: live confocal image of cells transfected with TFG-mCherry-FLAG. Right panel: widefield deconvolution image of cells transfected with

FLAG-TFG-SNAP and incubated with SNAP 647-SiR. Scale bar 5 µm. **c** Widefield deconvolution microscopy of HeLa cells co-transfected with mGFP-Sec16L and FLAG-TFG-SNAP. Left panel: Cell overview. Scale bar 10 µm. Right panel: magnification of TFG condensate; individual channels and merged image are given. Scale bar 5 µm.

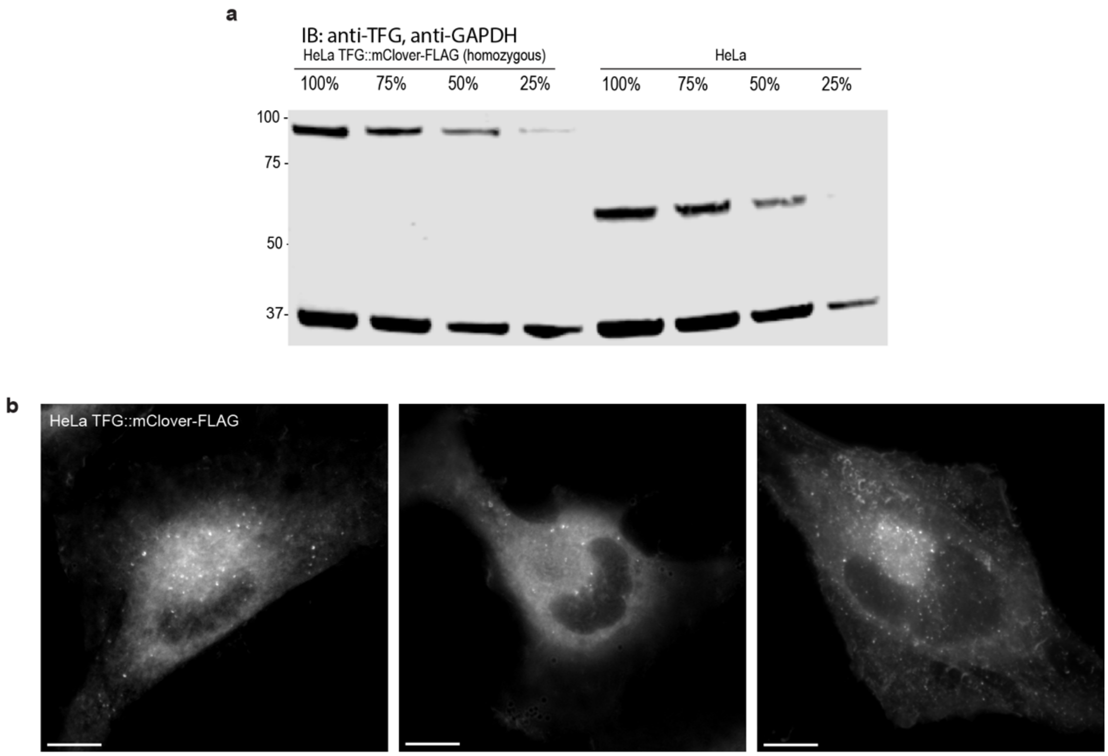

Supplementary Fig. 5. Homozygous endogenously tagged TFG::mClover-FLAG HeLa cells exhibit small, spherical, hollow condensates a Anti-TFG Western blot of HeLa TFG::mClover-FLAG cells (left) and wild type HeLa cells (right). 100%, 75%, 50%, 25% of samples loaded from lysates containing ~3 million cells. **B** Examples of HeLa cell clones harboring endogenously tagged TFG::mClover-FLAG. Scale bar 10 µm. Source data are provided as a Source Data file.

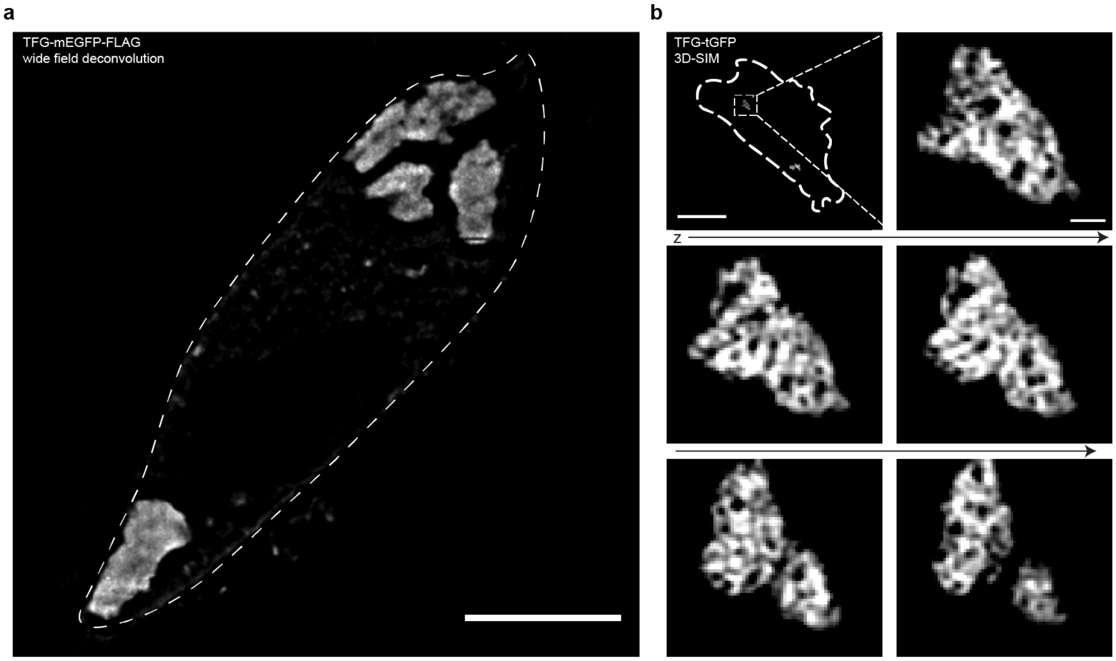

Supplementary Fig. 6. TFG condensates exhibit an anisotropic distribution in cells. A Live-cell widefield deconvolution microscopy of HeLa cells transfected with TFG-mEGFP-FLAG. Scale bar 10 µm. **B** 3D-Structured Illumination Microscopy (3D-SIM) of HeLa cells transfected with TFG-tGFP. Cell overview is provided (thick dashed line indicates cell outline), and serial sections are depicted. Z-step = 125 nm. Scale bar 10 µm. Magnification scale bar 500 nm.

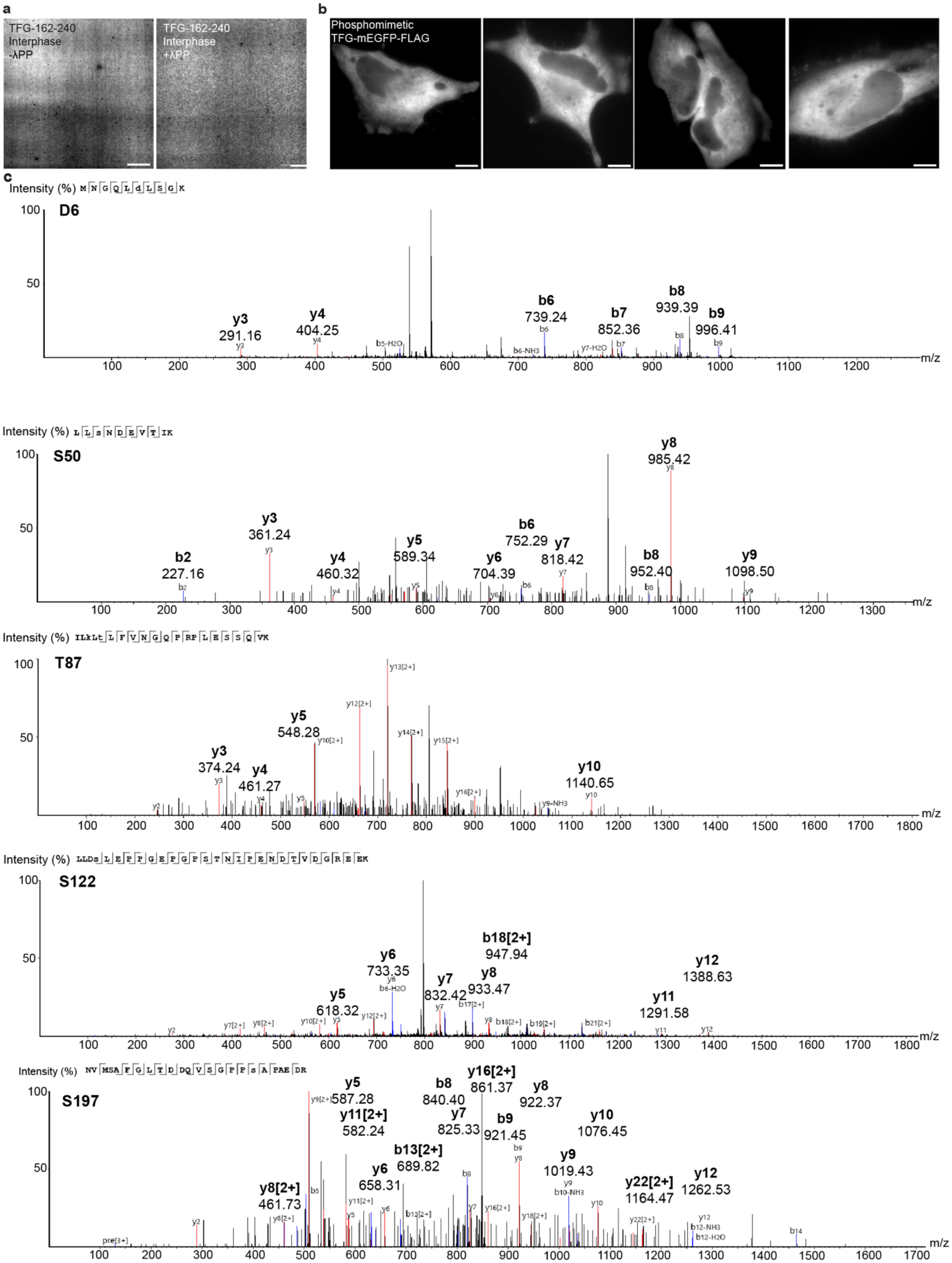
a
TFG-162-240 Interphase -λPP
TFG-162-240 Interphase +λPP
b
Phosphomimetic TFG-mEGFP-FLAG
c
Intensity (%)
D6
y3 291.16
y4 404.25
b6 739.24
b7 852.36
b8 939.39
b9 996.41
m/z
S50
b2 227.16
y3 361.24
y4 460.32
y5 589.34
y6 704.39
b6 752.29
y7 818.42
b8 952.40
y8 985.42
y9 1098.50
T87
y3 374.24
y4 461.27
y5 548.28
y10 1140.65
S122
y5 618.32
y6 733.35
y7 832.42
y8 933.47
b18[2+] 947.94
y11 1291.58
y12 1388.63
S197
y8[2+] 461.73
y5 587.28
y11[2+] 582.24
y6 658.31
b13[2+] 689.82
b8 840.40
y7 825.33
y16[2+] 861.37
b9 921.45
y8 922.37
y9 1019.43
y10 1076.45
y22[2+] 1164.47
y12 1262.53

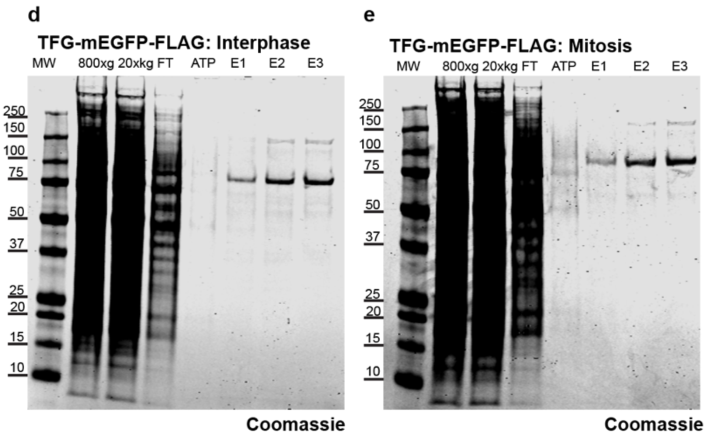

Supplementary Fig. 7. Cell cycle mediated condensation of TFG is regulated by phosphorylation. a Representative confocal micrographs of recombinant TFG-162-240-mEGFP-FLAG purified from synchronized interphase Expi293F which was concentrated below saturation and treated with lambda protein phosphatase where indicated. Scale bar 10 µm. **b** Gallery of micrographs of phosphomimetic TFG-mEGFP-FLAG 24 hours post-transfection. Scale bar 10 µm. **c** Mass spectrometry spectra of confidently identified phosphosites listed from top to bottom: D6, S50, T87, S122, and S197. Mass spectra are annotated to depict query masses identified. **d** Purification overview (Coomassie) of TFG-mEGFP-FLAG purified from interphase or **e** mitotic Expi293f cells. Protein used in Phos-Tag (Fig. 3d). Source data are provided as a Source Data file.

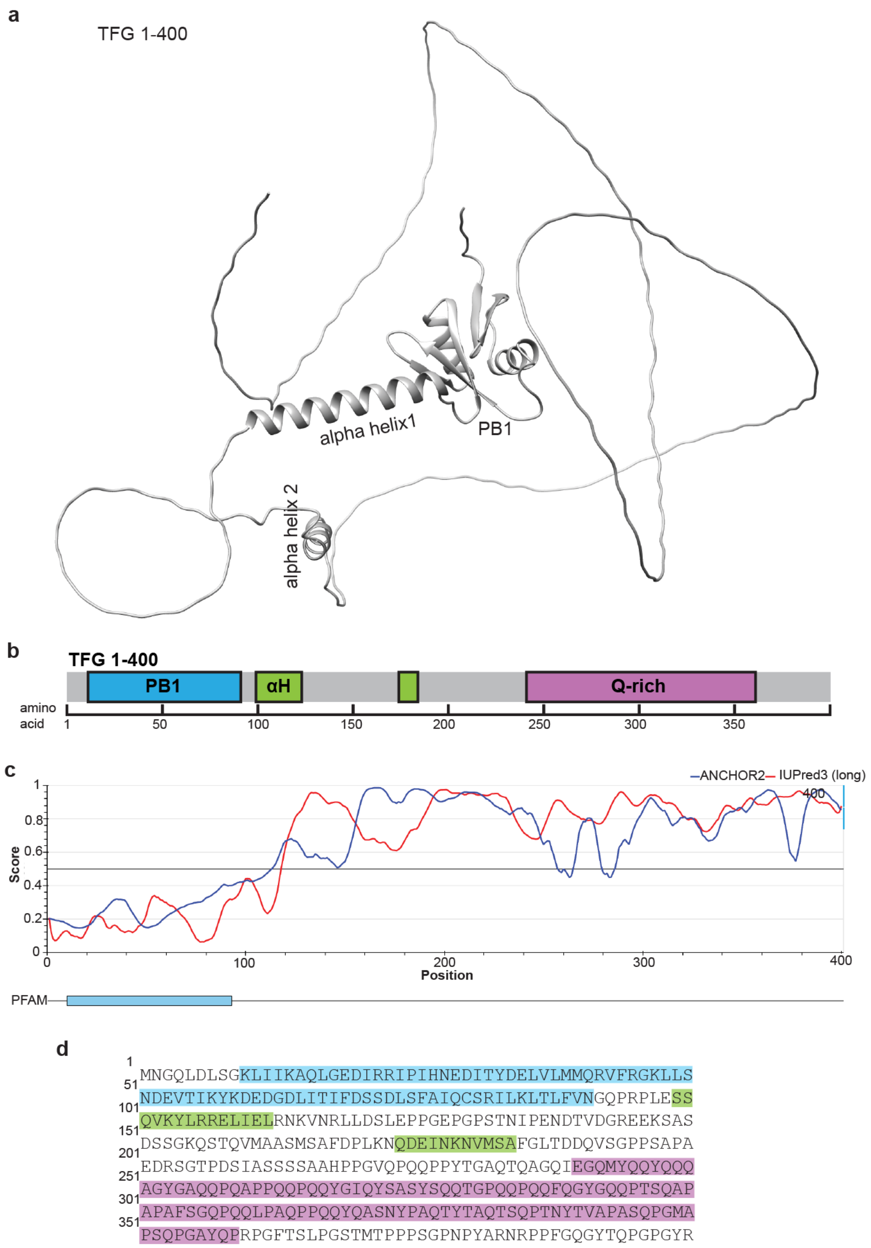

Supplementary Fig. 8. AlphaFold-predicted structure of TFG residues 1-400. IUPred predictions of full length TFG show regions of disorder. a Structure of TFG as predicted by AlphaFold with PB1, and high-confidence alpha helices 1 and 2 annotated. **b** Diagram of TFG domain structure (to scale). PB1 domain: blue, alpha helices: green, glutamine-rich region: purple. **c** IUPred3 predictions of TFG, with the red line symbolizing propensity for disorder in the structure and the blue line indicating the propensity to be part of a disordered binding region (58). **d** Amino acid sequence of TFG in rows of 50 amino acids, color-coded as in **b**.

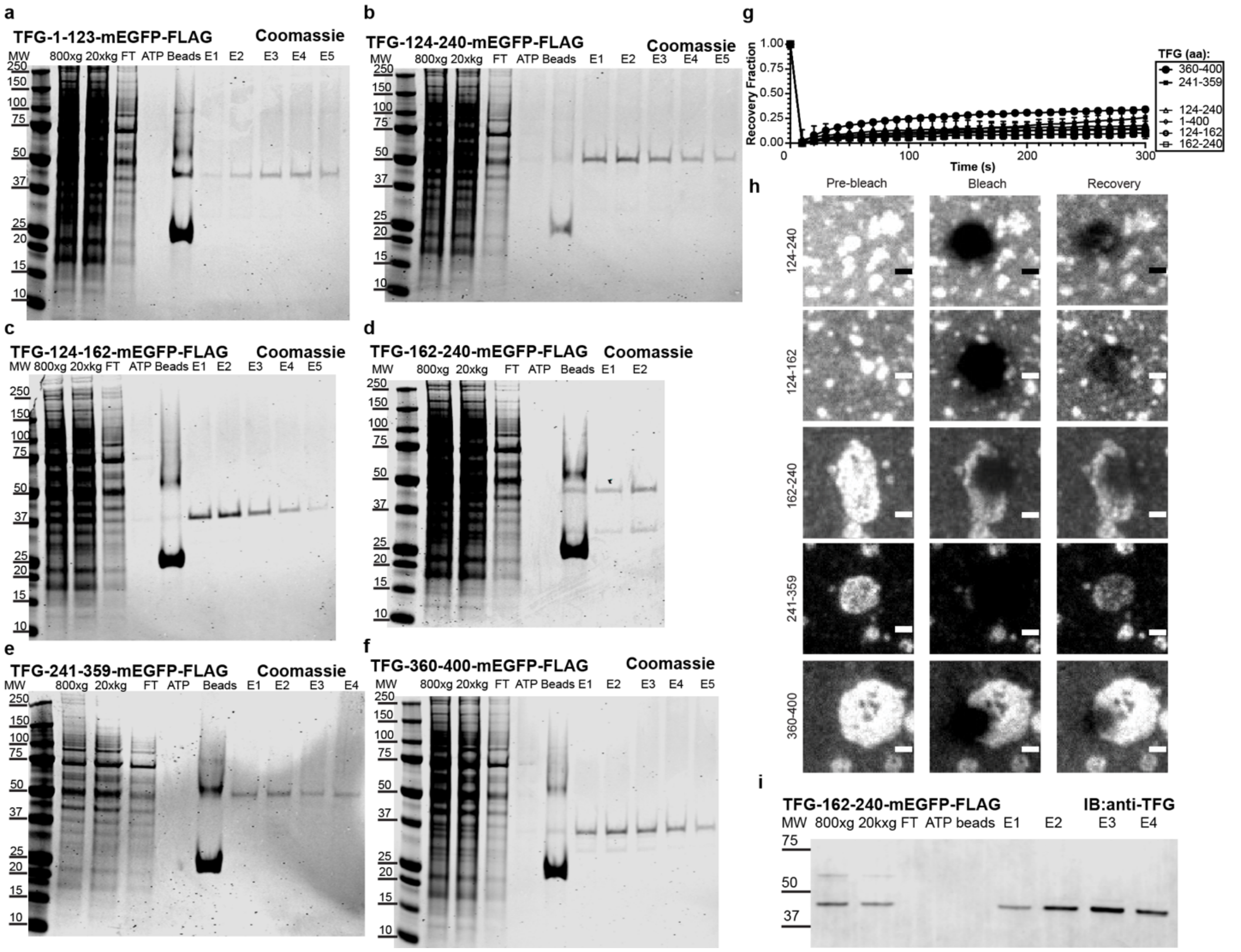

Supplementary Fig. 9. Purification overview and FRAP of truncated TFG constructs. Representative purification overview (Coomassie) of **a** TFG-1-123-mEGFP-FLAG. **b** TFG-124-240-mEGFP-FLAG. **c** TFG-124-161-mEGFP-FLAG. **d** TFG-162-240-mEGFP-FLAG. **e** TFG-241-359-mEGFP-FLAG. **f** TFG-360-400-mEGFP-FLAG. **g** FRAP curves of condensates formed by TFG truncations. TFG 1-400: n = 8; TFG 124-240: n = 11; TFG 124-161: n = 10; TFG 162-240: n = 10; TFG 241-359: n = 15; TFG 360-400: n = 4. Error bars are standard deviation. **h** Visual correlates for FRAP experiments of: TFG-124-240-mEGFP-FLAG, TFG-124-162-mEGFP-FLAG, TFG-162-240-meGFP-FLAG, TFG-241-359-mEGFP-FLAG, TFG-360-400-mEGFP-FLAG (HEPES/KOH pH 7.3; 150 mM KCl; 20% (v/v) PEG 8 kDa). Scale bars 1 µm. Larger, sponge-like condensates were selected to obtain partial FRAP data due to the size of individual condensates. **i** Representative anti-TFG Western blot for purification of TFG-162-240-mEGFP-FLAG. Source data are provided as a Source Data file.

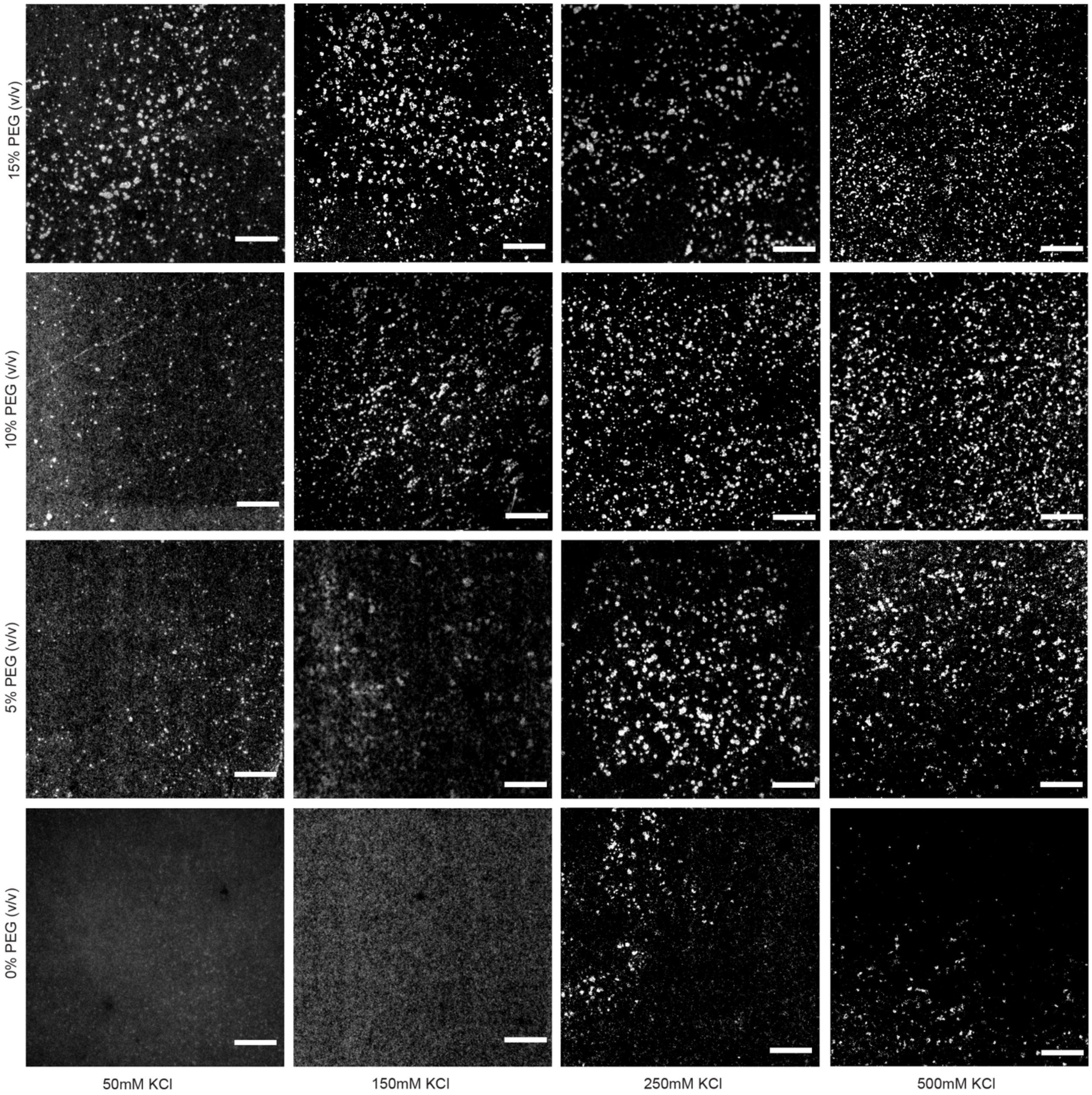

Supplementary Fig. 10. TFG phase diagram (visual correlates). Elevated buffer ionic strength promotes anisotropic condensation of TFG. TFG-mEGFP-FLAG: 70 nM; HEPES/KOH 7.3, X mM as indicated; X% (v/v) PEG 8 kDa as indicated. Scale bar 10 µm.

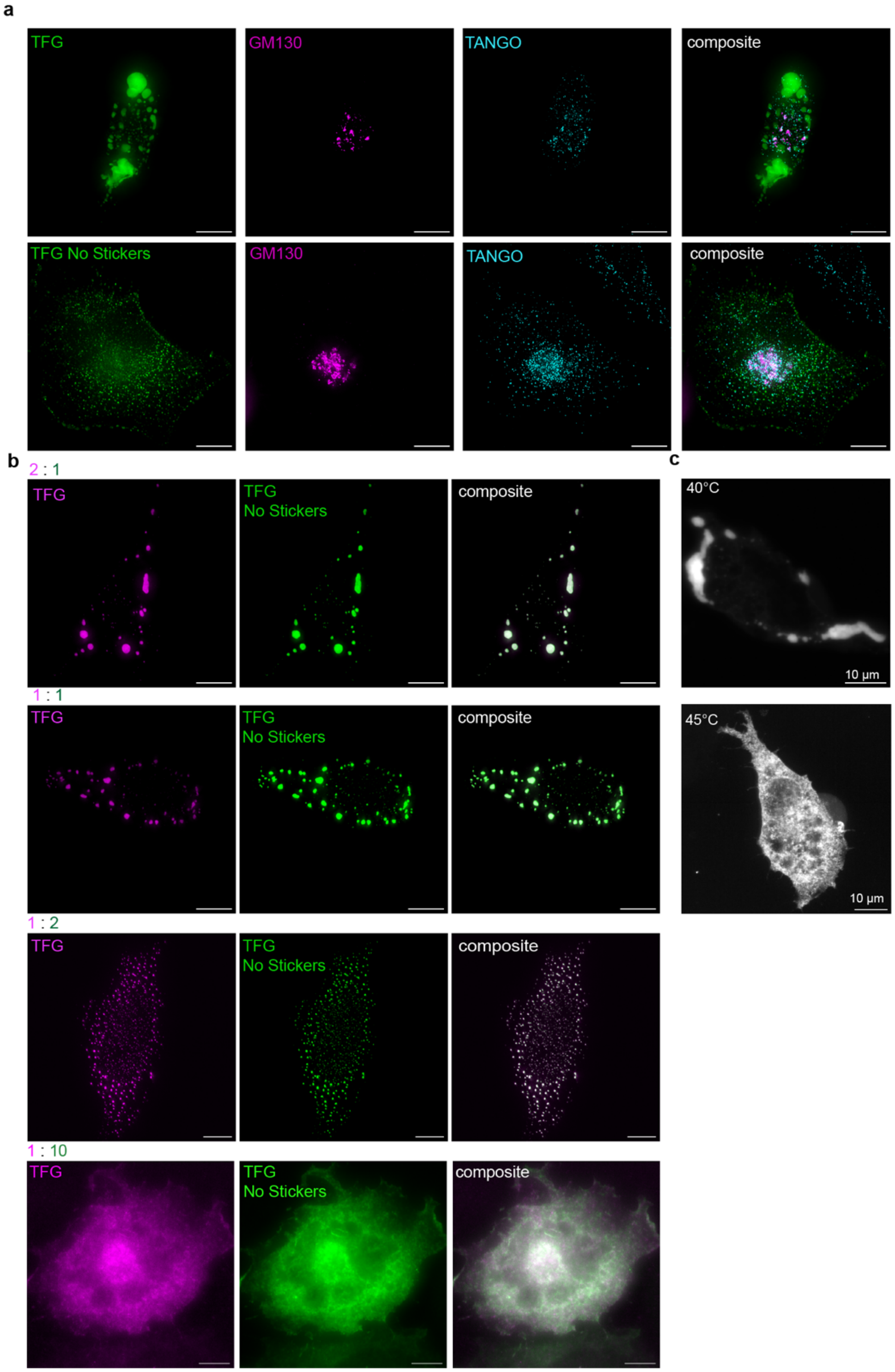

Supplementary Fig. 11. TFG disrupts endogenous ERES and Golgi markers only if 'sticker' residues are present. a Max. intensity Z-projections of HeLa cells transfected with TFG-mEGFP-FLAG or TFG-w/o 'stickers'-mEGFP-FLAG and immunolabeled with GM130 and TANGO1 exhibiting Golgi disruption without sticker residues. Scale bar 10 µm. **b** Max. intensity Z-projections of HeLa cells transfected with FLAG-TFG-SNAP (stained with SiR 647) and TFG-

w/o ‘stickers’-mEGFP-FLAG in ratios (as µg of plasmid DNA transfected per 100,000 cells) as indicated. Scale bar 10 µm. **c** Representative micrographs of HeLa cells transfected with TFG-mEGFP-FLAG shifted to the indicated temperatures. Scale bar 10 µm.

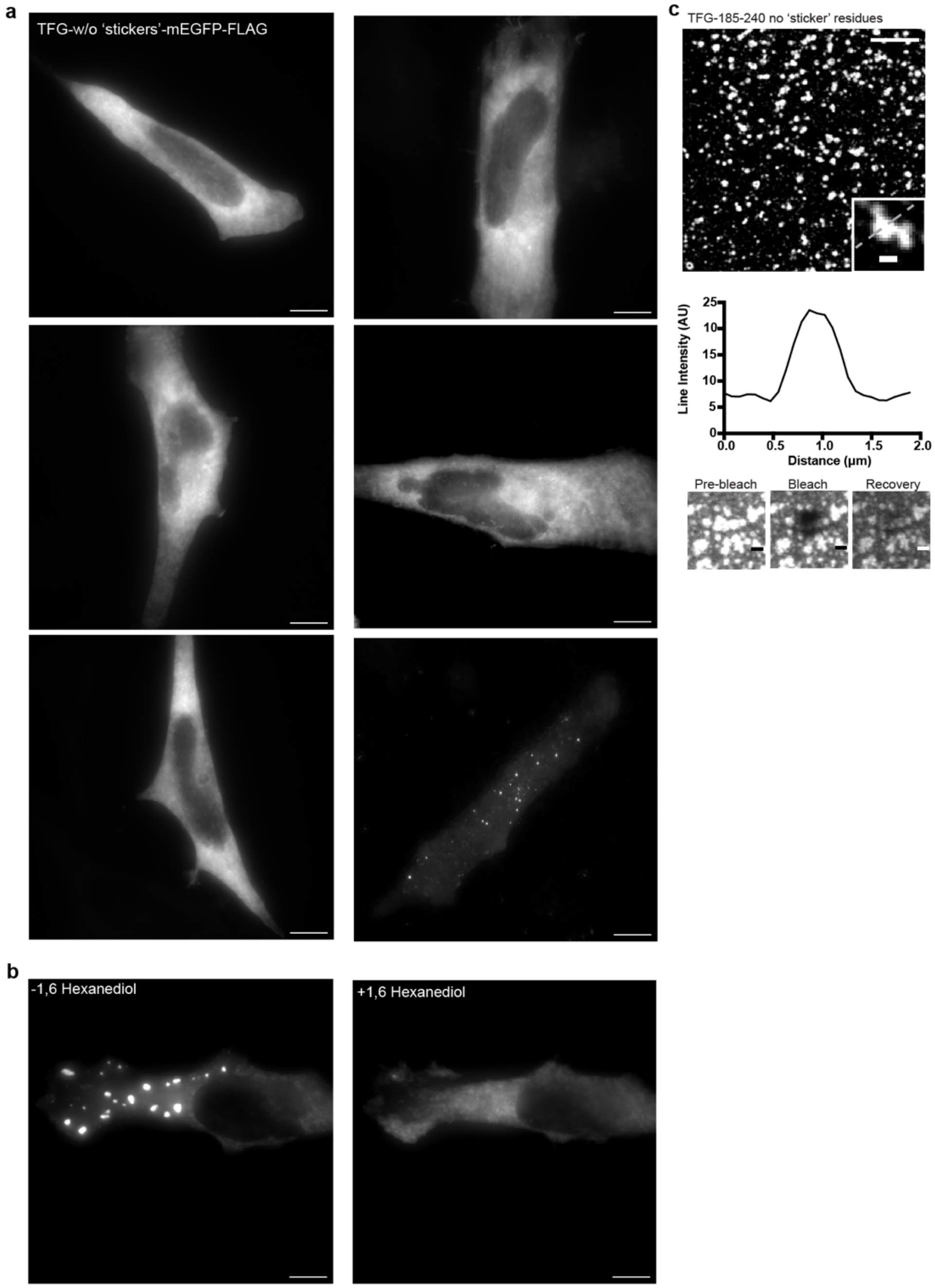

Supplementary Fig. 12. Anisotropic condensation of TFG is dependent on ‘sticker’ residues. **a** Gallery of micrographs of HeLa cells 24 hrs post-transfection of TFG-no ‘stickers’-mEGFP-FLAG exhibiting mostly cytosolic distribution and rarely small punctae. Scale bar 10 µm. **b** Micrograph of HeLa cells 24 hrs post-transfection of TFG-mEGFP-FLAG before and after 1,6-hexanediol addition, indicating sticker residues are critical for formation of condensates. Scale bar 10 µm. **c** Recombinant TFG-185-240-w/o ‘stickers’-mEGFP-FLAG (HEPES/KOH pH 7.3; 150 mM KCl; 20% (v/v) PEG 8 kDa). Scale bar 5 µm, 500 nm insets. Line intensity graph of

condensate, represented by dashed line, shows no lumen. FRAP of TFG-185-240-w/o 'stickers'-mEGFP-FLAG. Recovery t = 10 minutes. Scale bars 1 µm. Source data are provided as a Source Data file.

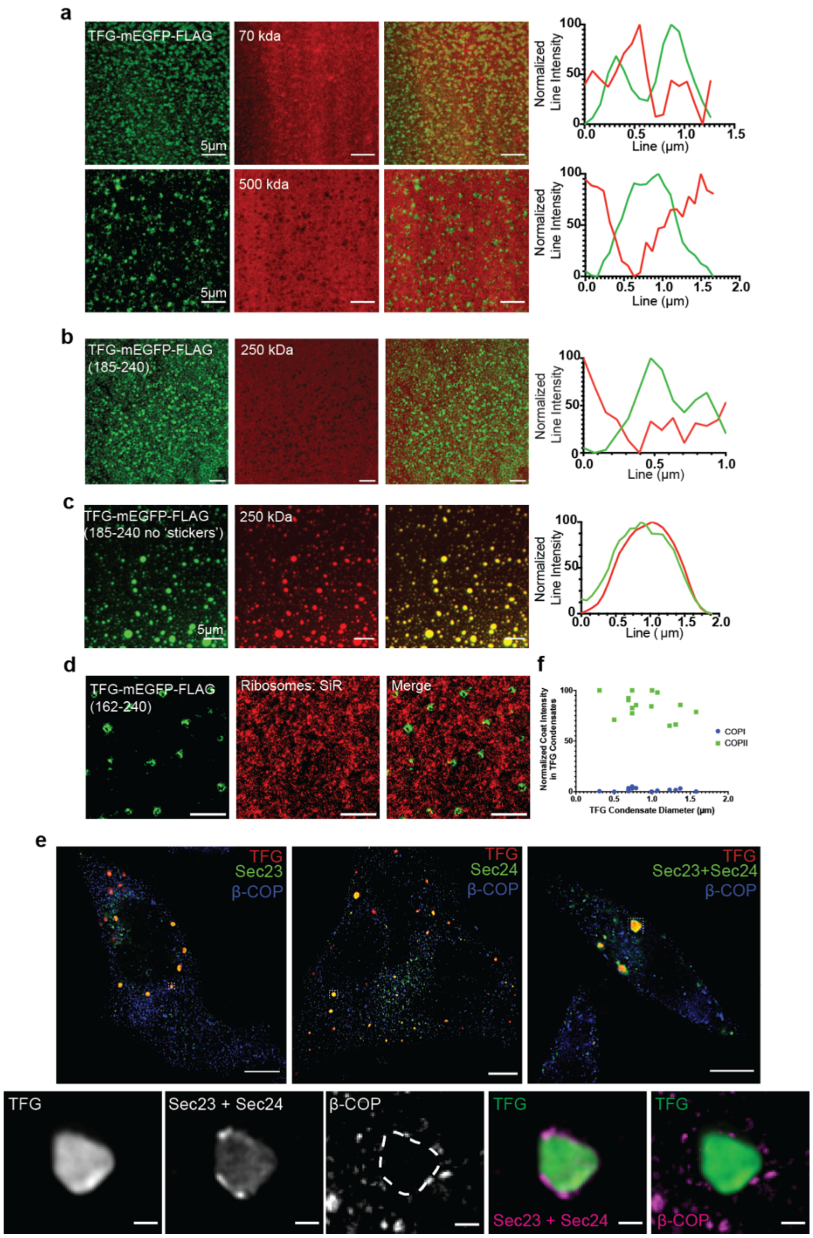

Supplementary Fig. 13. Visual correlates for TFG condensates inclusion and exclusion of dextrans of defined molecular weights, ribosomes, and COP components. a Recombinant protein condensates incubated for 10 minutes with dextran-TMR species as indicated. Normalized line intensities for representative images are given. Scale bars 5 µm. TFG-mEGFP-FLAG. Dextran-TMR species of 70 kDa and 500 kDa. Line scans depict TFG in green and dextran species in red. **b** TFG-185-240-mEGFP-FLAG. Dextran-TMR species of 250 kDa. **c** TFG-185-240-w/o 'stickers'-mEGFP-FLAG. Dextran-TMR species of 250 kDa. **d** TFG-162-240-mEGFP-FLAG incubated with NHS-SiR-labeled ribosomes. **e** Micrographs of FLAG-TFG-SNAP (stained with Snap-Cell 647 SiR), mEmerald-Sec23A, EYFP-Sec24C, and immunostained β-COP (as a proxy

for coatomer). Scale bar 10 μm (top), 1 μm (bottom). **f.** Mean gray value measurements of both Sec23-mEmerald and immunostained β-COP within the area of TFG condensates of varying sizes. Each marker was normalized to the highest fluorescent value within individual cells. Source data are provided as a Source Data file.

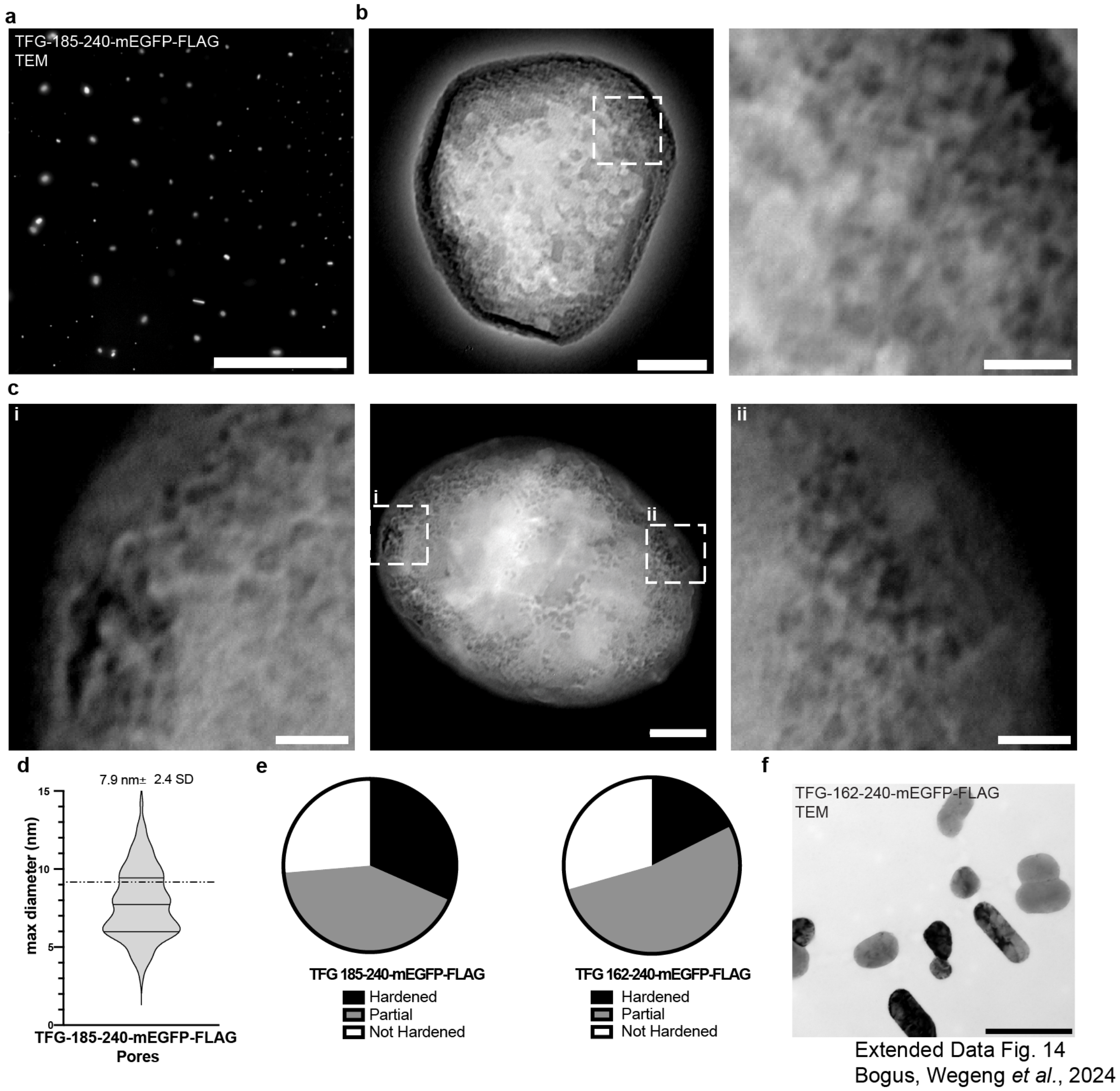

Supplementary Fig. 14. The alphahelical region (174-184) stabilizes TFG condensates but does not impact the condensation mechanism or alter condensate selectivity. a TEM overview of TFG-185-240-mEGFP-FLAG without negative staining. Scale bar 10 µm. **b** TEM micrograph of a single condensate of TFG-185-240-mEGFP-FLAG. Scale bar 100 nm. Magnification scale bar 25 nm. **c** TEM micrograph of a TFG-185-240-mEGFP-FLAG condensate. Scale bar 100 nm. Magnification scale bars: 25 nm. **d** Pore diameter quantification measured over the widest distance of pore. Dashed line represents average pore diameter of TFG 185-240-mEGFP-FLAG. Mean = 7.9 nm, standard deviation = 2.4 nm. n = 114 **e** Pie charts showing ratio of hardened, partially hardened, and not hardened condensates in TEM of TFG-185-240-mEGFP-FLAG (n = 19) and TFG-162-240-mEGFP-FLAG (n = 17) as indicated. **f** TEM overview of TFG-185-240-mEGFP-FLAG without negative staining. Scale bar 500 nm. Source data are provided as a Source Data file.
