# Supplementary material for: A hollow TFG condensate spatially compartmentalizes the early secretory pathway
Source: Nat Commun. 2025 Apr 19;16:3715. doi: 10.1038/s41467-025-59118-1 (PMC12009431; doi:10.1038/s41467-025-59118-1)
Supplement: Supplementary file 2 — Reporting Summary [file 41467_2025_59118_MOESM2_ESM.pdf]

nature portfolio

Corresponding author(s): Andreas M. Ernst

Last updated by author(s): Apr 4, 2025

Reporting Summary

Nature Portfolio wishes to improve the reproducibility of the work that we publish. This form provides structure for consistency and transparency in reporting. For further information on Nature Portfolio policies, see our Editorial Policies and the Editorial Policy Checklist.

Statistics

For all statistical analyses, confirm that the following items are present in the figure legend, table legend, main text, or Methods section.

n/a	Confirmed	
☐	☒	The exact sample size (n) for each experimental group/condition, given as a discrete number and unit of measurement
☐	☒	A statement on whether measurements were taken from distinct samples or whether the same sample was measured repeatedly
☐	☒	The statistical test(s) used AND whether they are one- or two-sided<br>*Only common tests should be described solely by name; describe more complex techniques in the Methods section.*
☒	☐	A description of all covariates tested
☒	☐	A description of any assumptions or corrections, such as tests of normality and adjustment for multiple comparisons
☐	☒	A full description of the statistical parameters including central tendency (e.g. means) or other basic estimates (e.g. regression coefficient) AND variation (e.g. standard deviation) or associated estimates of uncertainty (e.g. confidence intervals)
☐	☒	For null hypothesis testing, the test statistic (e.g. F, t, r) with confidence intervals, effect sizes, degrees of freedom and P value noted<br>*Give P values as exact values whenever suitable.*
☒	☐	For Bayesian analysis, information on the choice of priors and Markov chain Monte Carlo settings
☒	☐	For hierarchical and complex designs, identification of the appropriate level for tests and full reporting of outcomes
☒	☐	Estimates of effect sizes (e.g. Cohen's d, Pearson's r), indicating how they were calculated

Our web collection on statistics for biologists contains articles on many of the points above.

Software and code

Policy information about availability of computer code

Data collection	OMX Acquire SR (GE/Cytiva); softWoRx 7.2.2. (GE/Cytiva); LI-COR Image Studio 5.2; ColabFold v1.5.2-patch (AlphaFold2)
Data analysis	ImageJ 1.53c; AVIA Go 10.0.1 (Leica); Microsoft Office 365; GraphPad Prism 9 and 10; UCSF Chimera 1.16; LI-COR Image Studio 5.2

For manuscripts utilizing custom algorithms or software that are central to the research but not yet described in published literature, software must be made available to editors and reviewers. We strongly encourage code deposition in a community repository (e.g. GitHub). See the Nature Portfolio guidelines for submitting code & software for further information.

Data

Policy information about availability of data

All manuscripts must include a data availability statement. This statement should provide the following information, where applicable:

- Accession codes, unique identifiers, or web links for publicly available datasets
- A description of any restrictions on data availability
- For clinical datasets or third party data, please ensure that the statement adheres to our policy

All data that support the findings of this study are included within this paper and its extended data. Raw micrographs and analysis data are available upon request.

Research involving human participants, their data, or biological material

Policy information about studies with human participants or human data. See also policy information about sex, gender (identity/presentation), and sexual orientation and race, ethnicity and racism.

Reporting on sex and gender	n/a
Reporting on race, ethnicity, or other socially relevant groupings	n/a
Population characteristics	n/a
Recruitment	n/a
Ethics oversight	n/a

Note that full information on the approval of the study protocol must also be provided in the manuscript.

Field-specific reporting

Please select the one below that is the best fit for your research. If you are not sure, read the appropriate sections before making your selection.

[x] Life sciences [ ] Behavioural & social sciences [ ] Ecological, evolutionary & environmental sciences

For a reference copy of the document with all sections, see nature.com/documents/nr-reporting-summary-flat.pdf

Life sciences study design

All studies must disclose on these points even when the disclosure is negative.

Sample size	Sample sizes were not predetermined based on statistical methods. Sample sizes were chosen based on previously published methods that use the same assay type and gave rise to robust means and deviations (Rebane et al., 2020; Ziltener et al. 2020; Milanovic et al. 2018; Ernst et al. 2018).
Data exclusions	No data was excluded from our analyses.
Replication	All experiments were performed in at least three experimental replicates. All attempts of replication were successful.
Randomization	n/a
Blinding	n/a

Reporting for specific materials, systems and methods

We require information from authors about some types of materials, experimental systems and methods used in many studies. Here, indicate whether each material, system or method listed is relevant to your study. If you are not sure if a list item applies to your research, read the appropriate section before selecting a response.

Materials & experimental systems

n/a	Involved in the study	
[ ]	[x]	Antibodies
[ ]	[x]	Eukaryotic cell lines
[x]	[ ]	Palaeontology and archaeology
[x]	[ ]	Animals and other organisms
[x]	[ ]	Clinical data
[x]	[ ]	Dual use research of concern
[x]	[ ]	Plants

Methods

n/a	Involved in the study	
[x]	[ ]	ChIP-seq
[x]	[ ]	Flow cytometry
[x]	[ ]	MRI-based neuroimaging

Antibodies

Antibodies used	anti-GM130 (BD Biosciences, cat # BDB610822), anti-SEC16 (Invitrogen, cat # PA552182), anti-TFG (Abcam, cat # 156866), anti-TANGO1 (Abcam, cat # 244506), conjugated Alexa-Fluor Plus-conjugated antibodies (Invitrogen, cat # A32727; # A32732; # A48255)
Validation	1. anti-TFG: reactive with recombinant, pure TFG in Western blot; detection of endogenous and overexpressed TFG-mEGFP in HeLa and EXPI293F; manufacturer: knockout validated.

2. anti-SEC16: detection of overexpressed recombinant Sec16-GFP; manufacturer: immunofluorescence.
3. anti-TANGO1: Western blot and immunofluorescence (manufacturer)
4. anti-GM130: detection of recombinant GM130 (Western blot, pure protein) and in cells overexpressing mEGFP-GM130 (Rebane et al. 2020); manufacturer: Western blot and immunofluorescence.

Eukaryotic cell lines

Policy information about cell lines and Sex and Gender in Research

Cell line source(s)	HeLa-CCL2: ATCC (www.atcc.org); EXPI293F: Thermo Fisher Scientific (cat #A145227)
Authentication	Cell morphology was evaluated via DIC microscopy; organelle morphology was evaluated via immuolabeling fluorescence microscopy (widefield/deconvolution)
Mycoplasma contamination	All cell lines used are negative for mycoplasma contamination.
Commonly misidentified lines (See ICLAC register)	*Name any commonly misidentified cell lines used in the study and provide a rationale for their use.*
